# An intelligent method and platform for obtaining lettuce canopy coverage

**DOI:** 10.3389/fpls.2026.1749000

**Published:** 2026-02-12

**Authors:** Hongbo Liu, Pan Zhang, Jishu Zheng

**Affiliations:** 1 Research Institute of Agricultural Engineering, Chongqing Academy of Agricultural Sciences, Chongqing, China; 2 College of Information and Electrical Engineering, China Agricultural University, Beijing, China

**Keywords:** artificial intelligence, computer vision, growth monitoring, hydroponic crops, multiple growth stages, semantic segmentation

## Abstract

The canopy characteristics of crops are essential aspects for assessing crop growth status and conducting phenotype analysis. As one of the key indicators to measure crop growth situation, accurate canopy coverage assessment can provide a strong foundation for crop growth and yield monitoring. Considering plant growth differences, this study investigated the statistical method for assessing canopy coverage using visual technology, focusing on lettuce as the research subject. Firstly, a multi-variety and multi-growth stage hydroponic lettuce image dataset was constructed, which lays a data foundation for the construction of a semantic segmentation model. Secondly, in order to ensure the precision of semantic segmentation, this study proposed a Channel-Axial-Spatial attention mechanism module from the perspective of feature enhancement. To satisfy the lightweight demands of practical model deployment, this study replaced the original backbone network of PSPNet with MobileNetv3, greatly reduced model complexity while minimizing model performance degradation. Finally, we developed a group lettuce canopy coverage acquisition system by employing Python in conjunction with PyQt5 and embedded the pre-trained models CAS-PSPNet and MobileNetv3-PSPNet into the system for effectiveness verification. By integrating the proposed attention mechanism module with PSPNet, the integrated model outperformed FCN, Unet, SegNet, Deeplabv3+, GCN, ExFusion, ENet, BiseNet, FusionNet, LinkNet, RefineNet, LWRefineNet, and PSPNet in semantic segmentation of lettuce plant groups, achieving a Mean Intersection over Union of 0.9832. The Mean Intersection over Union of PSPNet based on lightweight improvement is 0.9717, and the model size is 9.3M. The results show that the proposed semantic segmentation method can accurately capture the crop canopy coverage, offering a feasible solution for real-time crop growth monitoring.

## 1 Introduction

With the increasing adoption and promotion of smart agriculture, precise management and control of various agriculture tasks have emerged as top priority, Accurate monitoring of crop growth status plays a critical role in making precise decision for all aspects of agricultural production (Kamilaris and Prenafeta-Boldú, 2018; Kubota et al., 2019; Tian et al., 2021; Shu et al., 2023). the precise detection of growth indicators such as crop leaf area and canopy coverage serves a dual purpose: it not only provides good data support for crop growth models and yield estimation but also reflects the adaptability of crops to their specific environmental conditions (Chang et al., 2021; Mohammadi et al., 2021). However, traditional crop growth parameter estimation predominantly relies on manual measurement by agricultural technicians, resulting in a significant labor input and time consumption, especially in large-scale cultivation scenarios (Keller, 2018; Lee et al., 2018; Zhang, 2020; Yoosefzadeh-Najafabadi et al., 2021). Therefore, achieving real-time, accurate, and automatic estimation of crop growth status in complex agricultural scenarios remains a pivotal challenge that needs to be solved in the agricultural production process.

Plant phenotyping is an emerging technology used for analyzing the external traits of crops such as shape, structure, size, color, etc. These traits result from the interplay of genotype and environment (Makanza et al., 2018; Xu et al., 2020). By combining artificial intelligence, machine vision and automation technologies, high-throughput, accurate and efficient plant phenotyping technologies have been rapidly developed, and these advancements find applications in crop breeding, nutrient monitoring, stress analysis, disease detection, freshness estimation, etc (Gou et al., 2018; Ma et al., 2018; Singh et al., 2018; Zhang et al., 2018a; Das et al., 2021, Das et al., 2022). In addition, a high-throughput plant phenotyping platform based on phenotyping technology can employ multiple sensors to measure important physical data of plants, such as structural characteristics, plant height, volume, color, fresh weight, wilting degree, flowers/fruits count, etc (Masuda, 2021). Among these, the visual sensor, as a key component of the phenotyping platform, can realize functions such as image information processing and feature extraction, which are characterized by low cost and rapid processing speed (Deery et al., 2021). Consequently, leveraging plant phenotyping technology enables accurate real-time estimation of crop growth status.

Nevertheless, plant phenotyping technology encounters challenges in real-world agricultural production scenarios, including its vulnerability to the complex background and environmental interference (Lee et al., 2018). Therefore, to achieve an accurate estimation of crop growth status, it is essential to separate the research object from intricate backgrounds and address the accurate segmentation of that object (Jiang et al., 2018). Traditional image segmentation methods mainly include techniques based on the region, threshold, graph theory, edge, energy functional, cluster analysis, wavelet transform, mathematical morphology, artificial neural network, and genetic algorithm (Fan et al., 2021; G. and Gopi, 2021; Mohammadi et al., 2021; Nikbakhsh et al., 2021; Quan et al., 2021a). Although there is no clear requirement for the size of the dataset, it still faces difficulties such as noise interference, uneven grayscale, parameter setting, filter selection, and low operation speed (Adams et al., 2020). Simultaneously, many traditional segmentation methods are tailored to specific images, making it challenging to achieve optimal segmentation results in dynamically changing complex environments many (Rangarajan and Purushothaman, 2020).

The application of information technologies, including artificial intelligence and computer vision, into the agricultural field provides a technical guarantee for accurate crop segmentation under complex environmental conditions (Guo et al., 2021). For example, Sadashivan et al (Sadashivan et al., 2021). proposed a fully automatic segmentation method based on artificial intelligence for UAV-captured images. The method is applied to five datasets, containing three different crops and different growth stages, to calculate the leaf area index. Yang et al (Yang et al., 2020). took the leaf images captured under complex backgrounds as the research object, using Mask R-CNN for leaf segmentation model and VGG16 for leaf classification model. The results show that the average misclassification error of the segmentation model is 1.15%, and the accuracy of the classification model is 91.5%. Zhu et al (Zhu et al., 2020). proposed an automatic segmentation approach for field corn, including skeleton extraction, skeleton-based coarse segmentation, and stem-and-leaf classification-based fine segmentation. Their results reveal an impressive average accuracy of 0.964, which applies equally well to fully unfolded leaves and wrapped leaves. To monitor the growth, size, and yield of plants, Trivedi and Gupta (Trivedi and Gupta, 2021) proposed an automatic monitoring method based on U-Net. The method achieved accuracies of 94.91%, 94.93%, and 95.05% on the training dataset, validation dataset, and test dataset, respectively. Bhagat et al (Bhagat et al., 2022). introduced a new leaf segmentation and counting method Eff-ENet++ by combining EfficientNet-B4 and UNet++ with the goal of segmenting individual plant leaves. This method realizes the accurate extraction of image features through EfficientNet-B4, and improves the reliability of the segmentation algorithm. Lu et al (Lu et al., 2022). presented a new method for robust automatic plant segmentation in color images, which enhanced both plant and background segmentation by unconstrained optimization, utilizing a linear combination of RGB three-channel images. Chang et al (Chang et al., 2021). embedded artificial intelligence methods, such as segmentation models into some smart greenhouse systems. These systems enable real-time monitoring of growth parameters such as leaf number, leaf area, and dry matter mass. The results demonstrate the feasibility and efficiency of using artificial intelligence modeling in agriculture-related tasks. Marani et al (Marani et al., 2021). proposed a new method to improve cluster pixel segmentation for grape bundle. It compared the effects of pre-trained models including AlexNet, GoogLeNet, VGG16, and VGG19 in the segmentation process, among which VGG19 exhibited the most favorable performance, achieving an average segmentation accuracy of 80.58%. Notably, obtaining growth parameters such as leaf area through instance segmentation is only one aspect of AI segmentation methods. Zenkel et al (Zenkl et al., 2022). constructed a semantic segmentation model using DeepLab v3+ with high-resolution outdoor winter wheat datasets of different genotypes and growth stages. The IoU of plants and soil reached values of 0.77 and 0.90, respectively, outperforming regression support vector machines and random forest classification methods. Li et al (Li et al., 2022). proposed PlantNet, a dual functional segmentation network that can simultaneously achieve semantic segmentation and instance segmentation. Its performance was validated on tobacco, tomato, and sorghum datasets. The IoU of semantic segmentation reached 85.86%, while the average precision of instance segmentation reached 83.30%. Du et al (Du et al., 2023). reported the plant segmentation transformer network (PST) for point cloud data segmentation, which achieved a MIoU of 93.96% for semantic segmentation on the rapeseed plant dataset. Besides, by combining PST and PointGroup, the instance segmentation reached an average precision of 88.83%. In addition, segmentation can also facilitate the counting of flowers and fruits, as well as the acquisition of fruit phenotypic parameters (Zabawa et al., 2020; Li et al., 2021; Rahim et al., 2021). Li et al (Li et al., 2024). proposed a CNN-based Edge Enhancement Network (EENet), which strengthens the model’s ability to learn edge information during the training phase, thereby improving crop segmentation accuracy. Picon et al (Picon et al., 2025). innovatively introduced a semantic segmentation framework for crop condition assessment and conducted research on crop disease identification using semantic segmentation on a self-constructed dataset. Kong et al (Kong et al., 2024). proposed a novel segmentation network based on the YOLO architecture, enabling precise delineation of crop contours while avoiding additional computational resource demands. Cao et al (Cao et al., 2025). proposed a nearly unsupervised crop segmentation method termed DepthCropSeg, which leverages Depth Anything V2 to generate pseudo-labels and selects optimal label data for model training, substantially reducing the time investment required for data annotation. Li et al (Li et al., 2025). designed a multi-level knowledge distillation framework tailored for agricultural scenarios, integrating high-level semantic information with low-level texture features during distillation, which significantly enhanced segmentation accuracy in complex environments. Zheng et al (Zheng et al., 2024). proposed a lightweight semantic segmentation model based on UAV visible-light imagery for multi-crop classification, providing technical support for crop categorization and intelligent field monitoring via drones. Ye Mu et al (Mu et al., 2026). proposed a hybrid object semantic segmentation model PCSNet based on unmanned aerial vehicle remote sensing, mainly used for segmenting 14 crops and 2 backgrounds. The mIoU of the model reached 84.61%. Gangwar et al (Gangwar et al., 2025). proposed a tomato leaf disease segmentation model called Tomato TransDeepLab, which can not only recognize leaf diseases but also quantify the severity of diseases. Kang et al (Kang et al., 2025). developed an integrated IoT and computer vision monitoring system for crop growth management. The system utilizes a recursive image segmentation model to process sequential images for continuous monitoring of crop growth, and has been validated for feasibility on cabbage. Guerra Ibarra et al (Guerra Ibarra et al., 2025). compared the effects of different backbones on UNet segmentation performance and identified a backbone network VGGNet suitable for precise segmentation of leaves, fruits, and backgrounds. Zhao et al (Zhao et al., 2025). proposed a field lettuce segmentation model YOMASK, which achieved a segmentation accuracy of 95.41% by improving the feature extraction and fusion of the network itself.

Existing research has achieved relevant results in the field of crop segmentation, but there are still the following limitations. (1) Research on crop segmentation in large-scale field planting scenarios is mostly focused on disease classification, crop classification, and other aspects. There is relatively little research on crop growth supervision, especially in small-scale facility planting scenarios. (2) Most existing research focuses on the construction of segmentation models and growth parameter statistics for specific growth stages or individual leaves, with relatively few studies on the entire growth stage and population crops. (3) There are relatively few intelligent platforms for crop segmentation and growth parameter statistics, and research and algorithm implementation are not closely related.

Therefore, this study takes hydroponic lettuce as the research object, fully considering the differences in variety and growth stage, and constructs a population lettuce semantic segmentation model from 2 perspectives: model performance improvement and model lightweight deployment. Then, growth monitoring and statistical analysis of multiple varieties of lettuce at different growth stages are carried out. The specific contributions are as follows:

1. A semantic segmentation dataset was constructed for multiple varieties of lettuce at different growth stages, taking into account the phenotype and growth differences of different lettuce varieties.
2. To ensure the segmentation accuracy of the model, this study proposes a new attention mechanism CAS, which effectively improves the segmentation reliability of PSPNet.
3. To meet the lightweight requirements of model deployment, this study compared the improvement effects of different lightweight backbone networks on PSPNet and found that PSPNet based on MobileNetv3 achieved a basic balance between model size and model precision.
4. A semantic segmentation model suitable for multiple varieties of lettuce and an intelligent platform for obtaining canopy coverage have been developed, effectively improving the efficiency of obtaining key growth parameters for lettuce populations.

## 2 Materials and methods

### 2.1 Experimental field

**Figure 1:**
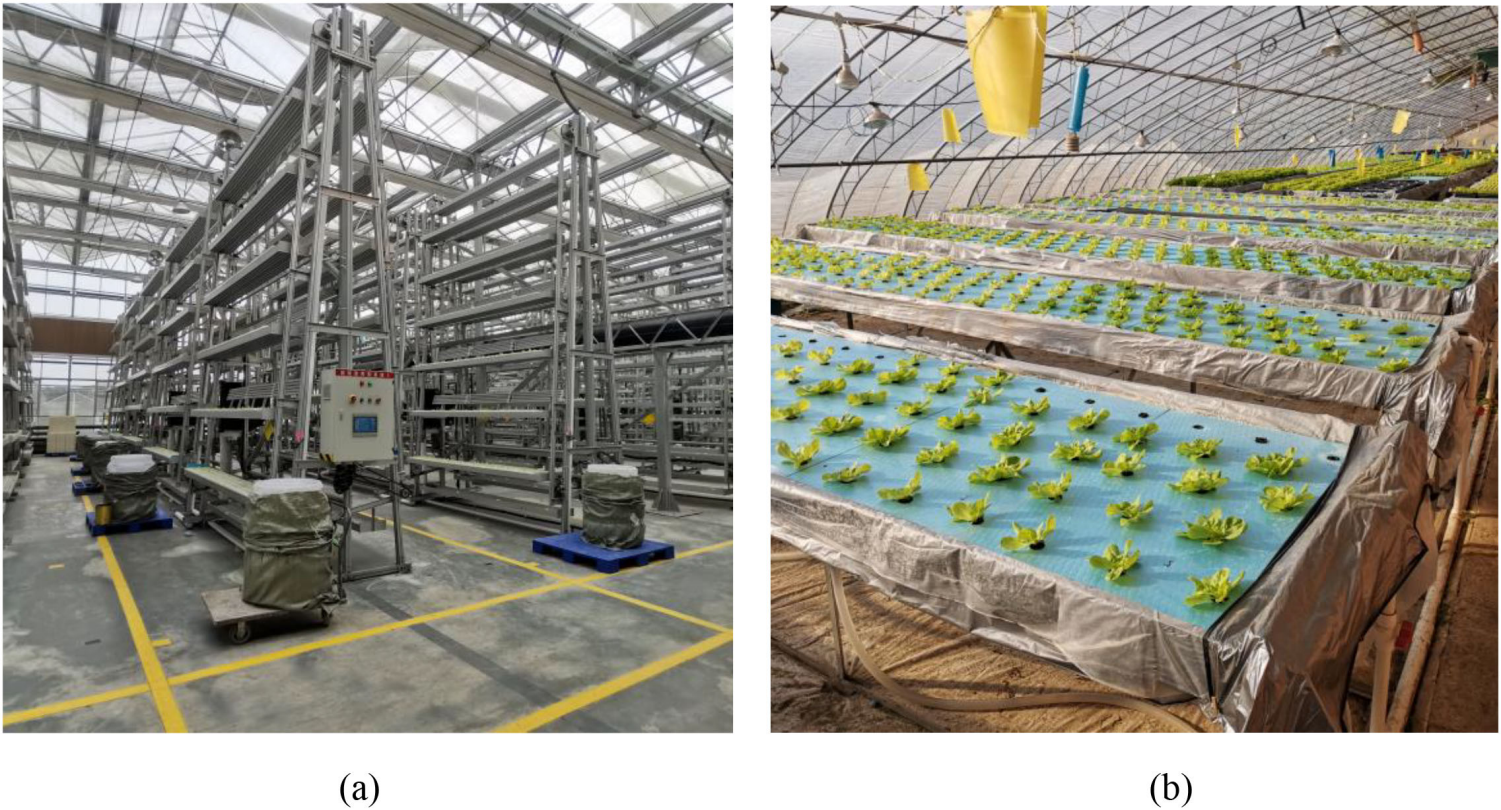
Experimental scenarios in Chongqing and Beijing. **(a)** represents the stereoscopic cultivation in Chongqing, and **(b)** represents the flat cultivation in Beijing.

Considering that difference in regions, planting patterns, etc. may lead to differences in the growth status of the same crop, experiments were carried out in both Chongqing and Beijing from March to May and October to December 2024. The planting scenarios are shown in **Figure 1**. In this experiment, the dataset was mainly collected from three varieties of hydroponic lettuce (*Small cream green*, *Batavia*, and *Boston cream*), named V1, V2, and V3. Images were collected after the lettuce were transplanted to hydroponic system.

### 2.2 Image acquisition

**Figure 2:**
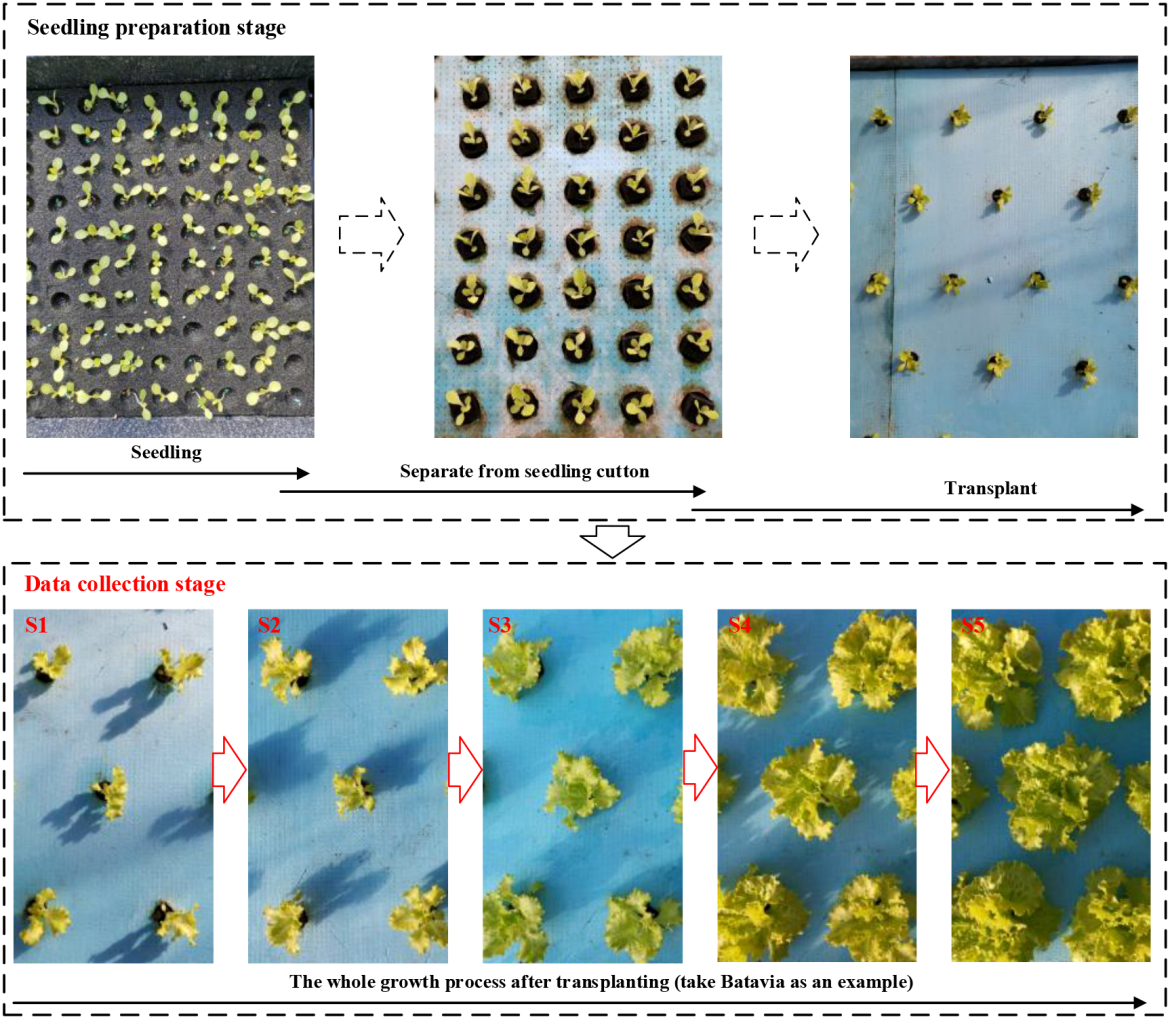
Data acquisition process.

**Figure 3:**
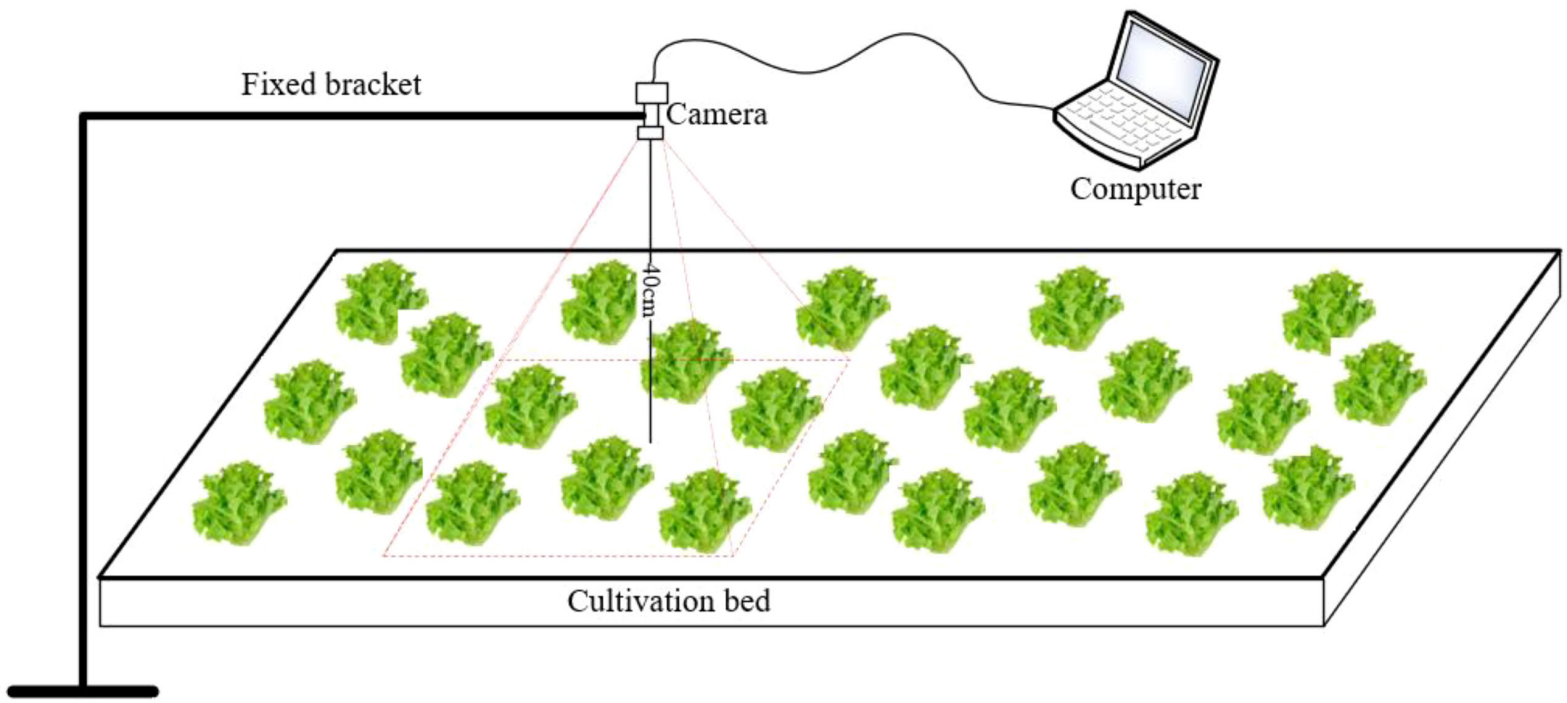
Image dataset acquisition.

In this study, there are differences in the cultivation environment between Chongqing and Beijing regions. Specifically, the temperature in Chongqing is relatively suitable from March to May, resulting in relatively fast growth rate of lettuce. Therefore, the data collection in Chongqing occurs every 5 days. While the growth rate of lettuce is slower due to the lower temperature in Beijing between October and December. Therefore, the data collection in Beijing occurs per 8 days. Following seedling transplantation (as shown in **Figure 2**), data collection in Chongqing occurred on the 5th, 11th, 17th, 23rd, and 29th days post-transplantation, with 100 images collected each time. Similarly, data collection took place in Beijing on the 9th, 18th, 27th, 36th, and 45th days after transplantation, with 100 images collected each time. 500 images were collected from the entire growth stage in Chongqing and Beijing, respectively, and totally 1000 images collected for model training. During the image data acquisition process, a fixed bracket is used to maintain the camera plane at a distance of 40 centimeters from the cultivation bed plane and parallel to it, to minimize data acquisition errors and ensure the reliability of subsequent result comparisons, as shown in **Figure 3**.

### 2.3 Image pre-processing

The acquisition of canopy coverage of the group lettuce based on the accurate segmentation of lettuce plants. According to the requirements of the segmentation network for the dataset, ***Labelme*** software was used to annotate lettuce plants in this study. In addition, considering the difference in the growth cycle of lettuce between Chongqing and Beijing, 500 images collected from Chongqing and Beijing were separately divided into training, validation and testing sets in a ratio of 6:2:2. Then, the same category of data sets from the two regions were summarized to create a model construction dataset with 600, 200 and 200 images for training, validation and testing respectively. Besides, during the model training process, we adjusted the image size to 640×640 pixels to meet the fundamental requirements of effective training.

### 2.4 Multi-strategy modeling methods

**Figure 4:**
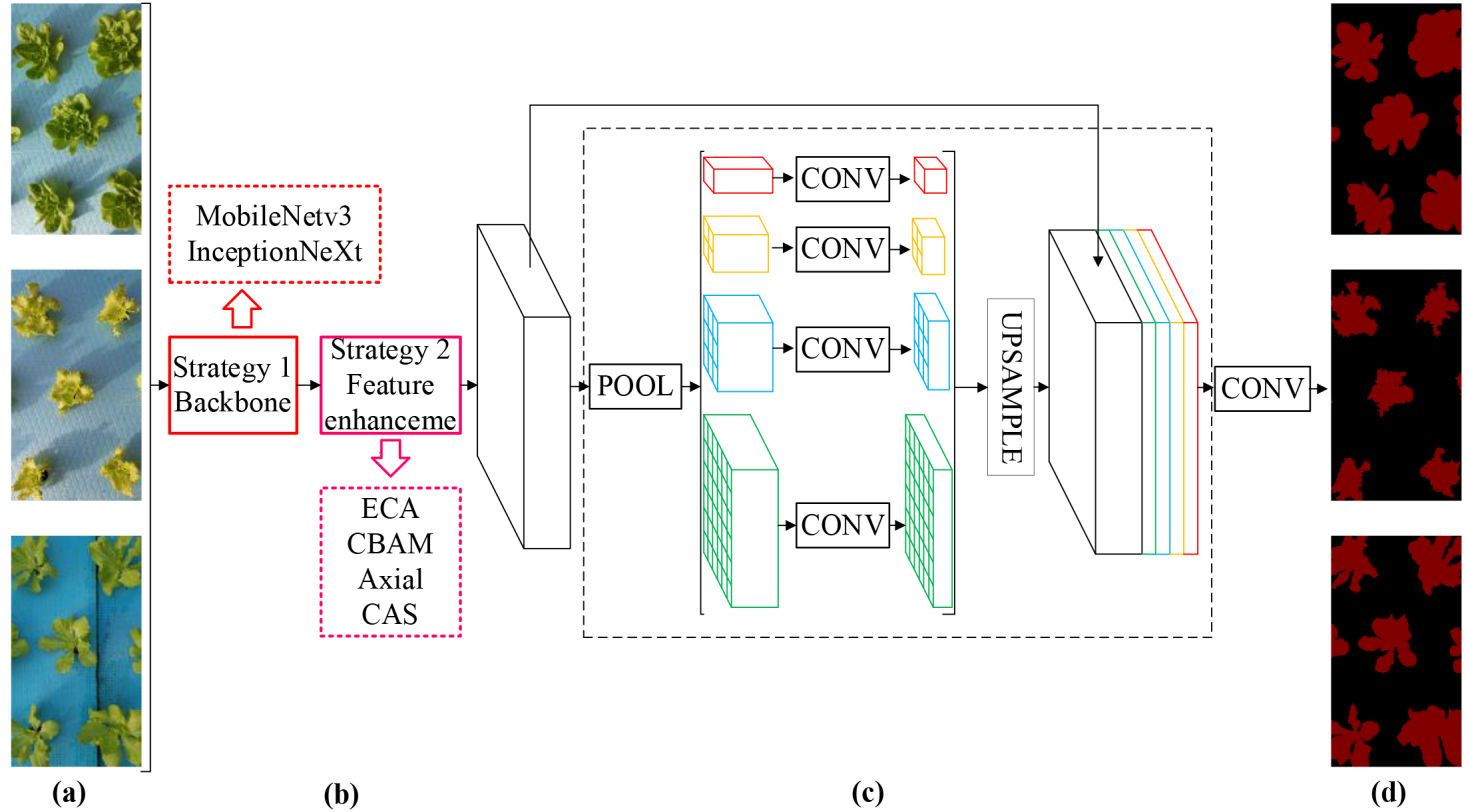
The network structure of the improved PSPNet. **(a)** Input image. **(b)** Feature map. **(c)** Pyramid pooling module. **(d)** Final prediction.

Crop development, from seedlings to mature plants, involves a dynamic growth process. It is prospecting to monitor the phenotypic changes of crops across different growth stages through machine vision technology to characterize their growth status. PSPNet is a pyramid scene parsing network (Zhao et al., 2017), addressing the limitation of full convolution networks by effectively integrating local and global feature information through a pyramid pooling module, thereby enhancing model precision. Notably, both the model reliability and the requirements of lightweight deployment in practical applications needs to be considered. Therefore, this study proposed CAS-PSPNet and MobileNetv3-PSPNet, focusing on both performance enhancement and model lightweighting. It enables accurate determination of the canopy coverage of the group lettuce in different growth stages, laying a good foundation for the growth monitoring and analysis of lettuce throughout its entire growth cycle. The improvement strategy for PSPNet is shown in **Figure 4**.

#### 2.4.1 Input data

This study mainly constructs the semantic segmentation model for the group lettuce datasets of three varieties and five growth stages. On the premise of considering the computational capacity of GPU and the dataset characteristics, we randomly partition the datasets of different varieties and different growth stages into the train, validation, and test subsets. Specifically, the train dataset and validation dataset participate in the process of model construction, and the test dataset serves to evaluate the reliability of the model.

#### 2.4.2 Backbone

The backbone feature extraction network of PSPNet is ResNet50. In order to meet the lightweight requirements in the actual deployment process of the model, this study uses MobileNetv3 (Howard et al., 2017) and InceptionNext (Yu et al., 2023) lightweight backbone to replace ResNet50 to achieve network lightweight. Both MobileNetV3 and InceptionNext backbone networks offer advantage of lightweight, but their ways of achieving lightweight are slightly different. MobileNetv3 achieves lightweight through the introduction of NAS and SE attention mechanisms, while InceptionNext mainly achieves lightweight through the use of bottleneck layers and residual connections.

#### 2.4.3 Feature enhancement module

**Figure 5:**
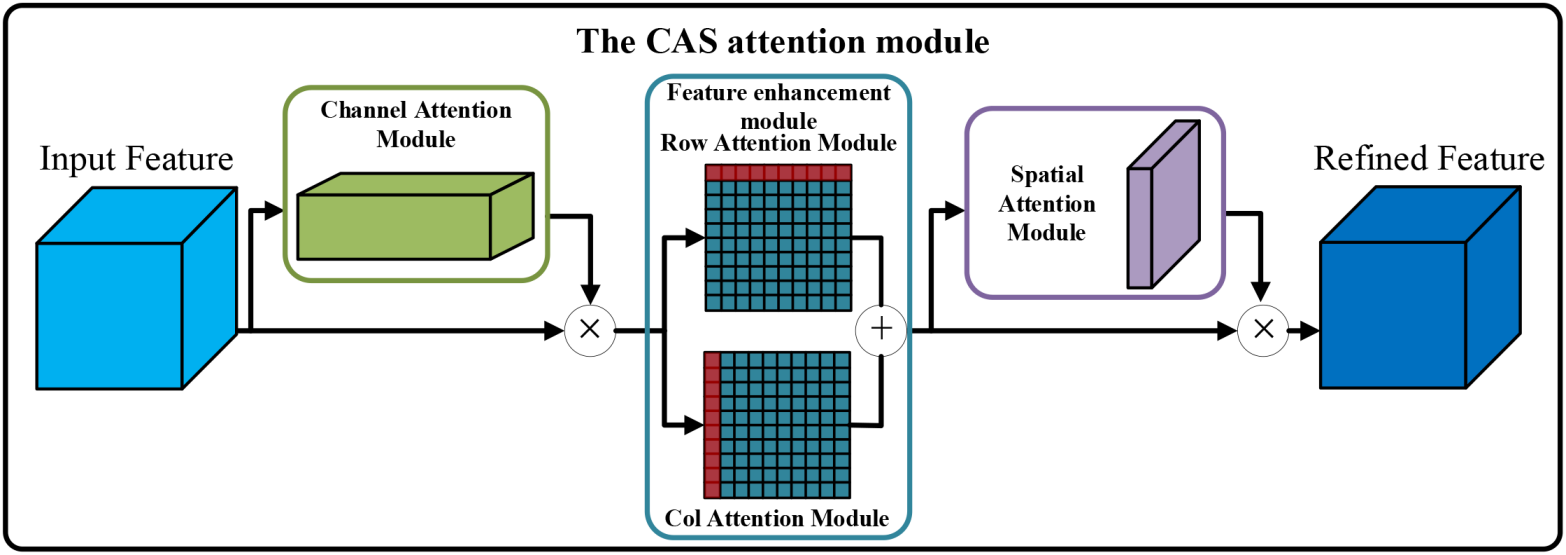
The network structure of the CAS attention mechanism.

To ensure the reliability of subsequent networks in pixel level classification, this study explored the enhancement effect of Axial (Ho et al., 2019), CBAM (Woo et al., 2018), and ECA (Wang et al., 2020) on the features from the backbone feature extraction network. Comprehensively consider the actual improvement effect of different attention mechanisms on the network, and be inspired by the feature enhancement method of Axial attention mechanism. On the premise of considering that CBAM enhances features in both dimensions of channel and spatial, this study introduced the row attention enhancement module and column attention enhancement module from the Axis attention mechanism to further enhance CBAM, and proposed an attention mechanism module called CAS. The CAS attention mechanism module is added to the end of the backbone feature extraction network to realize the secondary enhancement of plant features. The network structure of the CAS is shown in **Figure 5**.

Assuming that the feature F obtained from the backbone network generates two different spatial context descriptors $F_{avg}^{c}$ and $F_{\max}^{c}$ after entering the channel attention module. Then two spatial context descriptors are transmitted to a multi-layer perceptron (MLP) to generate channel attention feature ( MC(F)). This specific part mainly focuses on the network’s target, lettuce plants, by assigning a higher weight to the plant-related portion in the feature map processed by the module compared to the background. The specific calculation process is shown in Equation 1:

(1) $$\begin{array}{l} MC(F)=\sigma (MLP(AvPool(F)))+MLP(MaxPool(F))⊗F\\ =\sigma (W_{1}(W_{0}(F_{avg}^{c})))+W_{0}(W_{1}(F_{\max}^{c}))⊗F \end{array}$$

Where, σ represents the sigmoid function, $W_{0}$ and $W_{1}$ are the weights of MLP, $F_{avg}^{c}$ represents the average pooling feature, and $F_{\max}^{c}$ represents the maximum pooling feature.

In the actual planting scene, due to the interference of external environment such as light, the plants have certain color deviation. To ensure the reliability of subsequent segmentation, inspired by the feature enhancement concept of Axis attention mechanism, we made the following improvements following the channel attention module. Based on the channel attention feature map *MC* obtained above, input it into the row attention mechanism ( $R_{f}$) and column attention mechanism ( $C_{f}$) respectively to obtain the row feature and column feature (Details of $R_{f}$ and $C_{f}$ can be found in (Ho et al., 2019)), and then add the row feature and column feature as the input feature of the next module. The specific calculation process is shown in Equation 2.

(2) $$A(F)=R_{f}(MC)+C_{f}(MC)$$

Where, A(F) represents the output feature after the feature enhancement module.

Finally, input the feature A(F) after the secondary enhancement into the spatial attention module to generate a spatial attention feature map, guiding the network where the target object is, that is, the spatial information of lettuce plants. Subsequently, the MaxPool and AvgPool operations are performed on A(F) in turn, and the two are connected to generate effective feature descriptors ( $F_{avg}^{s}$ and $F_{\max}^{s}$). Then these descriptors are fed into the convolution layer to generate the spatial attention feature map ( MS(F)). The specific calculation process is shown in Equation 3.

(3) $$\begin{array}{l} MS(F)=\sigma (f^{7\times 7}([AvgPool(F);MaxPool(F)]))\\ =\sigma (f^{7\times 7}([F_{avg}^{s};F_{\max}^{s}])) \end{array}$$

Where, σ represents the sigmoid function, $f^{7\times 7}$ represents a convolution with the kernel size of 7 × 7.

#### 2.4.4 Pyramid pooling module

Traditional fully convolutional networks rely on local receptive fields and lack understanding of the overall structure of the image, resulting in category confusion when segmenting large-sized objects or complex scenes. PPM utilizes parallel processing of features at different scales to enable the model to simultaneously focus on local details and global context, thereby improving the segmentation accuracy and boundary clarity of targets at different scales.

The PPM is proposed and used to construct the global scene on the last layer feature map (*F*) of the ResNet50 network. This module mainly carries out feature fusion across four different scales (1×1, 2×2, 3×3, 6×6). By employing pooling operation, the global information of feature maps at different scales can be preserved, thereby better learning contextual information and ensuring the segmentation effect of the model. The specific calculation process is shown in equation X.

Assuming the k-th pooling branch is Equation 4:

(4) $$P_{k}=Upsample(Conv(AdaptiveAvgPool_{S_{k}}(F)))$$

Among them, $S_{k}\in \left\{1,2,3,6\right\}$. The final output feature is Equation 5:

(5) $$F_{out}=Conv_{1\times 1}(Concat(F,P_{1},P_{2},P_{3},P_{4}))$$

### 2.5 Model training and evaluation

This study is mainly completed based on RTX3090 (24G). In addition, to make better use of the pre-trained model for model construction, we use the combination of freeze and unfreeze for model training. Once the semantic segmentation model of the group lettuce plants of multiple varieties and growth stages is constructed, it is necessary to verify the model. At present, the commonly used evaluation indicators of semantic segmentation models include Pixel accuracy (*PA*) and Mean intersection over Union (*MIoU*). In addition, *Model-size*, *FPS*, and *Time-consuming* are also considered as the evaluation indicators to further evaluate the reliability of the model. The specific calculation process of some indicators shown in Equation 6–8.

(6) $$PA=\frac{TP+TN}{TP+TN+FP+FN}$$

(7) $$IoU=\frac{TP}{TP+FP+FN}$$

(8) $$MIoU=\frac{\sum_{i=1}^{C}IoU_{i}}{C}$$

where *FP, TP*, *TN*, and *FN* represent false positive, true positive, true negative, and false negative, respectively, and *C* represents the total number of categories.

## 3 Results

### 3.1 Comparison of semantic segmentation methods

To solve the problem of accurate semantic segmentation of lettuce in complex agricultural scenes, this study compared a total of 16 typical semantic segmentation methods to select the best modeling approach. The evaluated methods include: FCN (Shelhamer et al., 2017), UNet (Olaf et al., 2015), SegNet (Badrinarayanan et al., 2017), Deeplabv3+ (Chen et al., 2018), GCN (Peng et al., 2017), ExFuse (Zhang et al., 2018b), ENet (Paszke et al., 2016), BiseNet (Yu et al., 2018), FusionNet (Quan et al., 2021b), LinkNet (Chaurasia and Culurciello, 2018), RefineNet (Lin et al., 2017), LWRefineNet (Nekrasov et al., 2019), PSPNet (Zhao et al., 2017), Swin-transformer (Liu et al., 2021), SegNeXt (Guo et al., 2022), and DDRNet (Pan et al., 2023), The specific modeling results are shown in **Table 1**.

**Table 1:** Comparative analysis of modeling methods.

Class	PA	MIoU	Time	FPS	Model-size
FCN	0.9799	0.9516	0.0177s	56.5998	76.7 M
Unet	0.9855	0.9670	0.0323s	30.9489	51.1 M
SegNet	0.9765	0.9435	0.0193s	51.7139	92 M
Deeplabv3+	0.9755	0.9417	0.0209s	47.8321	210 M
GCN	0.9809	0.9558	0.0259s	38.5975	223 M
ExFuse	0.9841	0.9629	0.0294s	34.0559	329.06 M
ENet	0.9821	0.9583	0.0299s	33.4135	1.58 M
BiseNet	0.9801	0.9516	0.0103s	97.5374	47.4 M
FusionNet	0.9825	0.9576	0.0444s	22.5355	311 M
LinkNet	0.9772	0.9454	0.0107s	93.5670	44.1 M
RefineNet	0.9828	0.9580	0.0325s	30.7601	450 M
LWRefineNet	0.9826	0.9576	0.0142s	70.3649	104 M
Swin-transformer	0.9846	0.9805	0.0379s	26.3731	685M
SegNeXt	0.9857	0.9755	0.0283s	35.3653	317M
DDRNet	0.9752	0.9630	0.0120s	83.2541	156M
PSPNet	0.9907	0.9810	0.0201s	49.7709	187 M

As shown in **Table 1**, the above 16 methods performed well on the lettuce plant datasets of multiple varieties and different growth stages collected in this study. Each method exhibits distinct advantages within this dataset. By comparing the various evaluation indicators, it can be found that lightweight semantic segmentation models such as FCN, SegNet, BiseNet, LinkNet, and LWRefineNet, demonstrate slightly lower PA and MIoU than PSPNet, while offer smaller model sizes and better FPS performance than PSPNet. These differences are attributed to the lightweight model reducing complexity of network and computational by adjusting the depth or width of the network. However, this trade-off also weakening the model’s ability to extract features, causing a decrease in the model segmentation precision. Meanwhile, striking a balance between model-size and precision remains a challenge in lightweight models design. For instance, the earlier adoption of down sampling strategy in the design of ENet network architecture resulted in spatial information loss, impacting its segmentation accuracy. UNet is a well-known method used in medical image segmentation, excels in edge segmentation precision. However, in this study, UNet faces challenges when segmenting plant stems due to their similarity to the background. In some cases, UNet misclassify stems as part of the background, resulting in low segmentation precision of the model. Focusing on expanding receptive fields and multi-scale feature fusion, methods such as Deeplabv3+, Exfuse, FusionNet, RefineNetextract features through deep convolution, feature fusion of different layers, skip connections, etc. to improve the segmentation precision of the model. But the model performance is slightly lower than PSPNet due to the high similarity between features of background and some plant stem, as well as relatively high model complexity. GCN is another semantic segmentation method based on graph convolutional networks. As the number of layer increases, the representation vectors of nodes tend to be consistent, leading to over smooth problems and high model complexity. Both the model performance and FPS are lower than PSPNet. PSPNet explicitly aggregates multi-scale global features through the PPM, while Swin Transformer relies on self-attention mechanism to indirectly capture long-range dependencies (which may be limited by local windows). SegNeXt and DDRNet focus more on local feature extraction, resulting in stronger segmentation consistency of PSPNet in complex scenes such as small objects and boundary blurred areas. Therefore, we selected PSPNet with better performance of the core evaluation indicators (*PA* and *MIoU*) on the collected datasets as the main semantic segmentation method in this study.

### 3.2 Modeling results for improving model performance based on attention mechanism

To ensure the reliable performance of semantic segmentation models in complex agricultural scenarios, this study explored the enhancement effects of several mainstream attention mechanisms (e.g., CBAM, Axial, ECA) on PSPNet, with the primary objective of improving the robustness of lettuce plant segmentation through feature enhancement. Furthermore, we integrated the attention mechanism module (CAS) proposed in this study with PSPNet and compared its improvement effects with those of other mechanisms. The specific results are shown in **Table 2**.

**Table 2:** Comparison of modeling results under different attention mechanism improvements.

Class	PA	MIoU	Time	FPS	Model-size
PSPNet	0.9907	0.9810	0.0201s	49.7709	187M
ECA-PSPNet	0.9910	0.9820	0.0194s	51.5002	187M
CBAM-PSPNet	0.9903	0.9806	3.0241s	33.0681	183M
Axial-PSPNet	0.9900	0.9787	2.9071s	34.3989	218M
CAS-PSPNet	0.9903	0.9832	4.1415s	24.1456	223M

As shown in **Table 2**, the segmentation effects of PSPNet combined with different attention mechanisms (ECA, CBAM, Axial, and CAS) were compared. The results indicate that PSPNet’s performance was further enhanced when integrated with ECA and CAS. The ECA attention mechanism enhances feature representation by introducing channel attention into convolution operations. This enables the network to capture inter-channel dependencies, adaptively adjust channel feature weights, better focus on important features, and suppress less relevant ones, thereby improving feature discriminability. Compared to the baseline PSPNet, ECA-PSPNet shows slight improvements in PA, MIoU, and FPS, with increases of 0.0003, 0.001, and 1.7293, respectively. However, since ECA primarily focuses on channel relationships, it may not fully leverage spatial information. In contrast, CBAM extracts both channel and spatial features to enhance the model’s expressive and generalization capabilities, though its efficiency may decrease for distantly related features. The Axial attention mechanism captures global context by decomposing attention calculations along different axes, thereby improving model performance. The CAS attention mechanism proposed in this study not only considers channel and spatial features but also employs row-wise and column-wise attention mechanisms for secondary enhancement of channel features. A key indicator of semantic segmentation effectiveness, MIoU, increased significantly by 0.0022 with CAS, demonstrating that the proposed mechanism can effectively improve segmentation performance for lettuce across multiple varieties and growth stages.

### 3.3 Modeling results based on model lightweight improvement

Constructing a semantic segmentation model for the group lettuce not only emphasizes the improvement of model performance, but also considers the lightweight deployment requirements of the model. On the basis of PSPNet, this study replaced the original backbone network ResNet50 with lightweight backbone networks MobileNetv3 and InceptionNeXt, respectively, to explore the lightweight improvement effect of the model. The specific modeling results are compared in **Table 3**.

**Table 3:** Comparison of Lightweight Improvement Results.

Class	PA	MIoU	Time	FPS	Model-size
PSPNet	0.9907	0.9810	0.0201s	49.7709	187M
MobileNetv3-PSPNet	0.9868	0.9717	0.0123s	81.3879	9.3M
InceptionNeXt-PSPNet	0.9686	0.9385	0.0159s	62.6672	94.7M

As shown in **Table 3**, MobileNetV3-PSPNet experiences a smaller performance loss compared to its pre-improvement state, while showing significant improvements in Time, FPS, and Model-size. In particular, the model size has been reduced by approximately 20 times, which greatly enhances inference speed and meets real-time requirements in practical applications. In contrast, InceptionNeXt-PSPNet exhibits a relatively large performance drop compared to its earlier version, even though its model size is only halved. Although InceptionNeXt also achieves relatively fast inference speed, its segmentation accuracy is slightly lower than that of MobileNetV3. This is because the complex and powerful structure of InceptionNeXt makes it more susceptible to interference from background features during feature extraction. As a result, some stem pixels of plants are misclassified as background pixels, leading to reduced segmentation precision. Furthermore, due to its smaller number of parameters, MobileNetV3 has a lower computational load and faster inference speed, giving it an advantage in tasks with high real-time demands. Additionally, the design of MobileNetV3 is more concise and efficient. It incorporates residual connections and lightweight attention mechanisms, enabling better learning of feature representations and contextual information, which ensures stronger performance in downstream tasks such as segmentation. Overall, considering both reliability and lightweight needs, MobileNetV3-PSPNet is more suitable for practical production environments.

### 3.4 Construction of a canopy coverage acquisition platform

**Figure 6:**
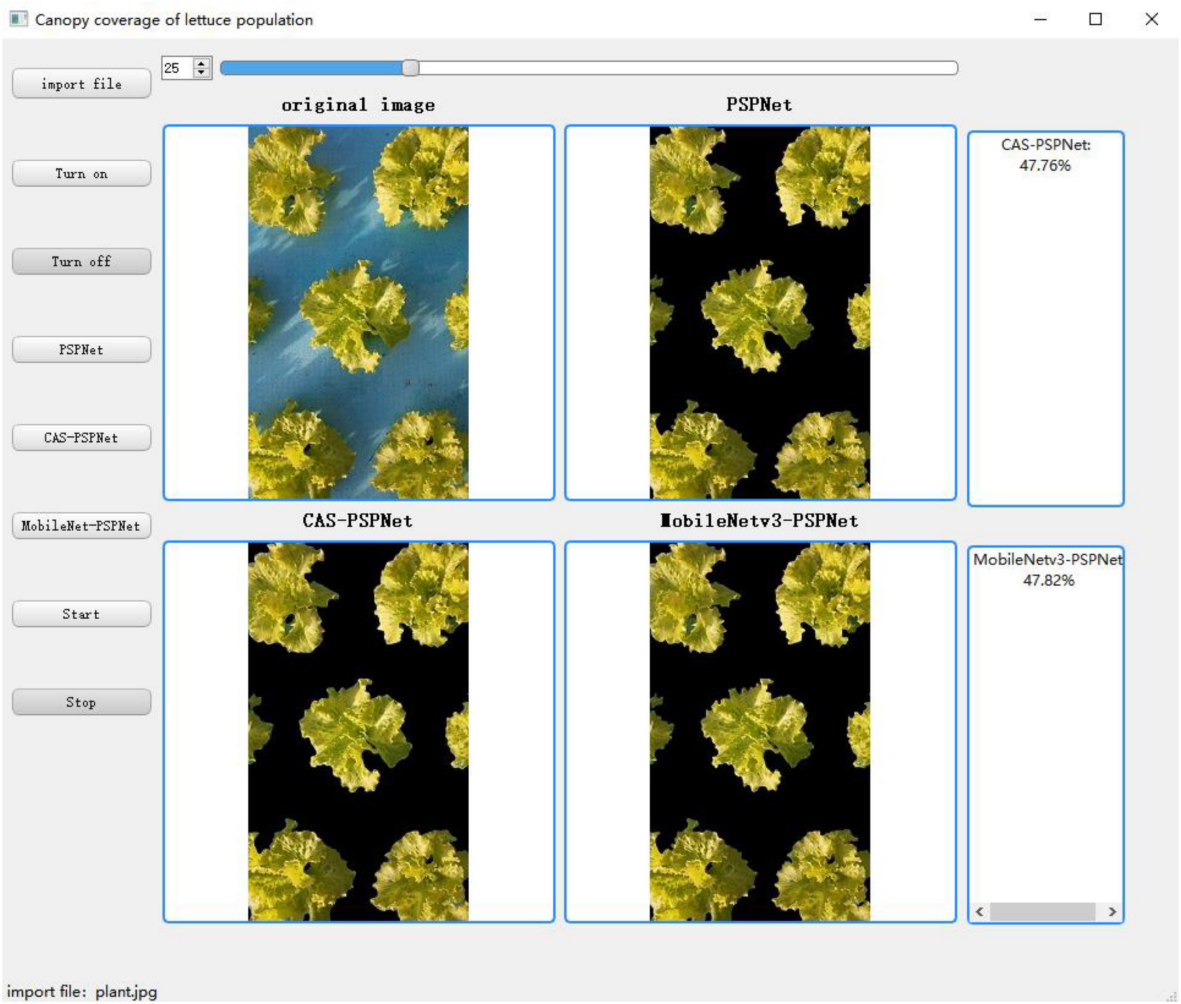
A system for obtaining canopy coverage of the group lettuce.

To facilitate the practical application of the aforementioned model for monitoring lettuce growth, this study developed a canopy coverage acquisition system for lettuce using Python and PyQt5. The entire system can supports both online or offline image data acquisition, weight file loading, and multi-model segmentation results acquisition. The specific system interface is shown in **Figure 6**.

As shown in **Figure 6**, the system is designed with four main functions for obtaining lettuce canopy coverage: threshold setting, image loading, pre-trained model loading, and result output. First, users can set the segmentation precision threshold using the slider at the top of the interface. Second, image or video data can be loaded by clicking the “Import File” or “Turn on” button on the left. Third, the corresponding pre-trained models (PSPNet, CAS-PSPNet, and MobileNetv3-PSPNet) are loaded by sequentially clicking their respective buttons. Finally, after clicking the “Start” button, the segmentation results from the selected model are displayed in the central area, while the calculated canopy coverage for the lettuce plants is shown in the display area on the right. The accuracy of canopy coverage calculation depends on the reliability of the semantic segmentation model. It is derived by calculating the ratio of plant-area pixels to the total image pixels in the model’s output. The system allows for the simultaneous output of segmentation results from both the baseline and improved models, as well as the canopy coverage information based on the CAS-PSPNet and MobileNetv3-PSPNet methods. This design facilitates a direct comparison between the two approaches.

### 3.5 Acquisition of canopy coverage of the group lettuce

**Figure 7:**
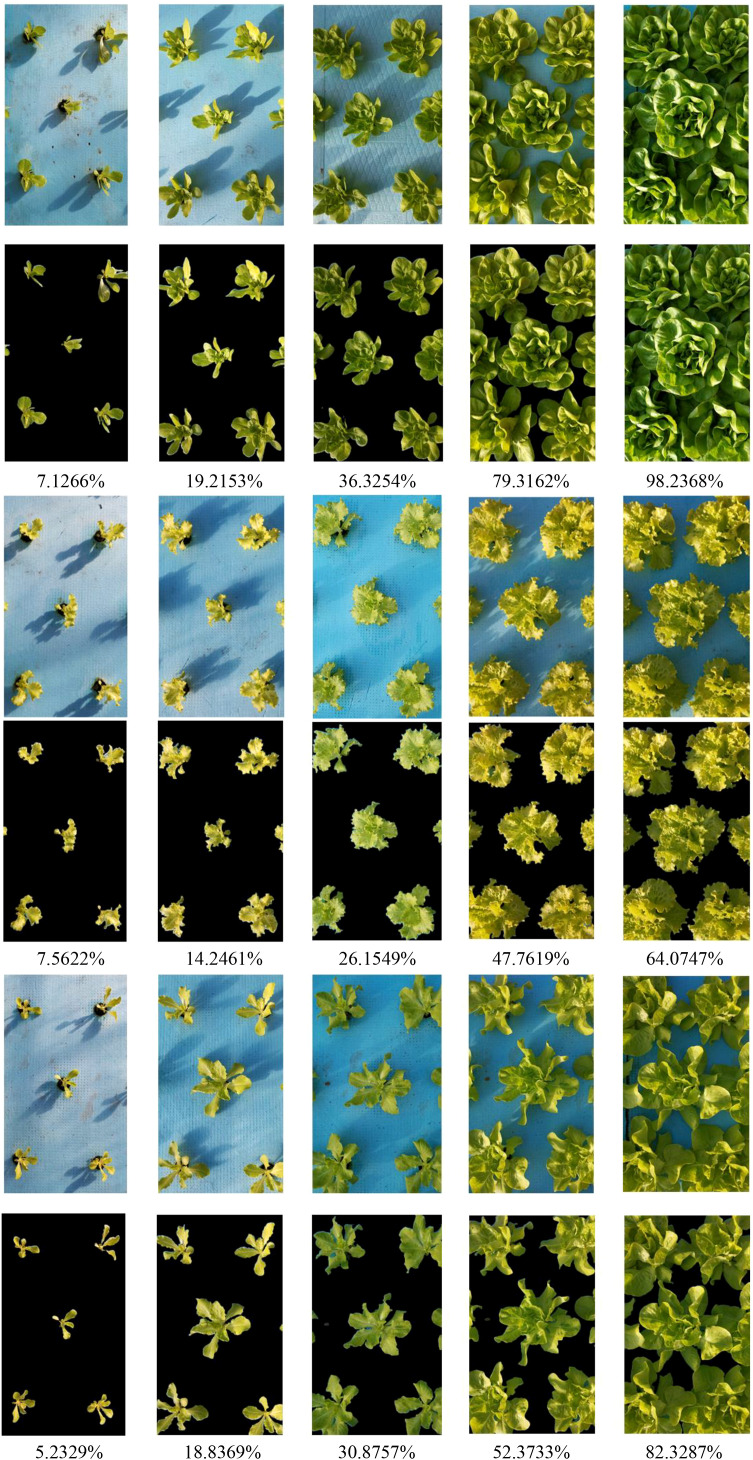
Acquisition of canopy coverage of the group lettuce in different varieties and growth stages.

In this section, we used the canopy coverage acquisition system (Section 3.4) to obtain canopy coverage data for lettuce plant across different varieties and growth stages. Taking the number of pixels as the basic statistical unit, calculating the proportion of pixels of the group plants within the whole image. This proportion was used as the measure of canopy coverage. The specific results are shown in **Figure 7**.

**Figure 8:**
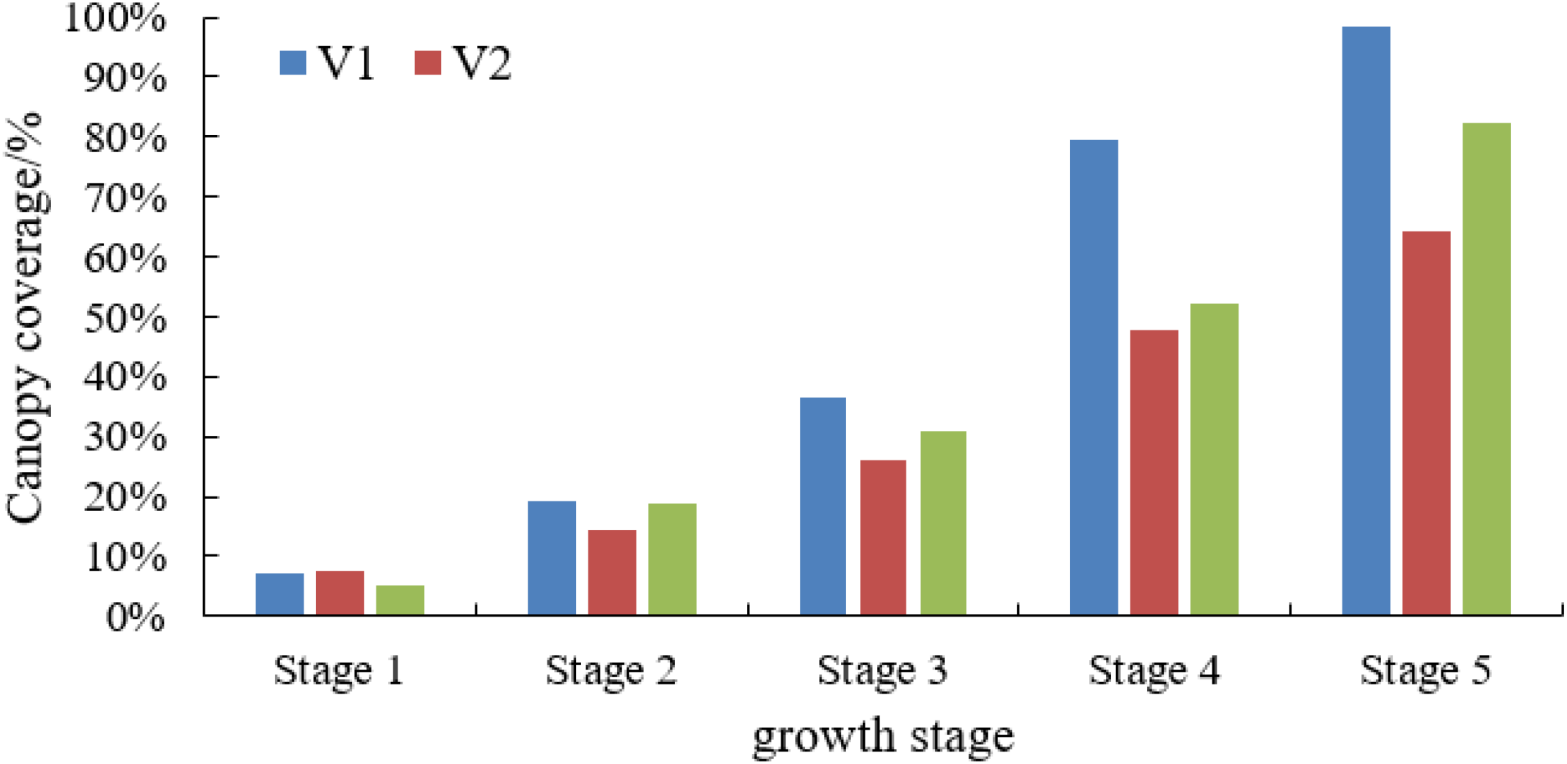
Canopy coverage of different lettuce varieties at different growth stages.

**Figure 7** shows the segmentation results and canopy coverage information of the group lettuce under different varieties and growth stages based on the CAS-PSPNet semantic segmentation method. It can be found that the model achieves a satisfactory segmentation performance, except for the minor background segmentation errors between leaves in the V3 variety in the last row. The canopy coverage information for lettuce of different varieties and growth stages, obtained through semantic segmentation models, can be used as an important indicator to measure the growth status of the group lettuce. This study also statistically analyzed the changes in canopy coverage of the group lettuce of different varieties and growth stages, as shown in **Figure 8**.

As shown in **Figure 8**, the canopy coverage information shows an increasing trend with the advancement of growth stage, and variations in canopy coverage are observed among different varieties of lettuce at different growth stages. The group lettuce canopy coverage acquisition system can be used in subsequent research for automatic calculation of the canopy coverage information of different crops and automatic analysis of the dynamic growth changes of different crop varieties from both quantitative and qualitative perspectives, which is certainly promising and applicable.

## 4 Discussion

### 4.1 Necessity of obtaining canopy coverage of the group plant

**Figure 9:**
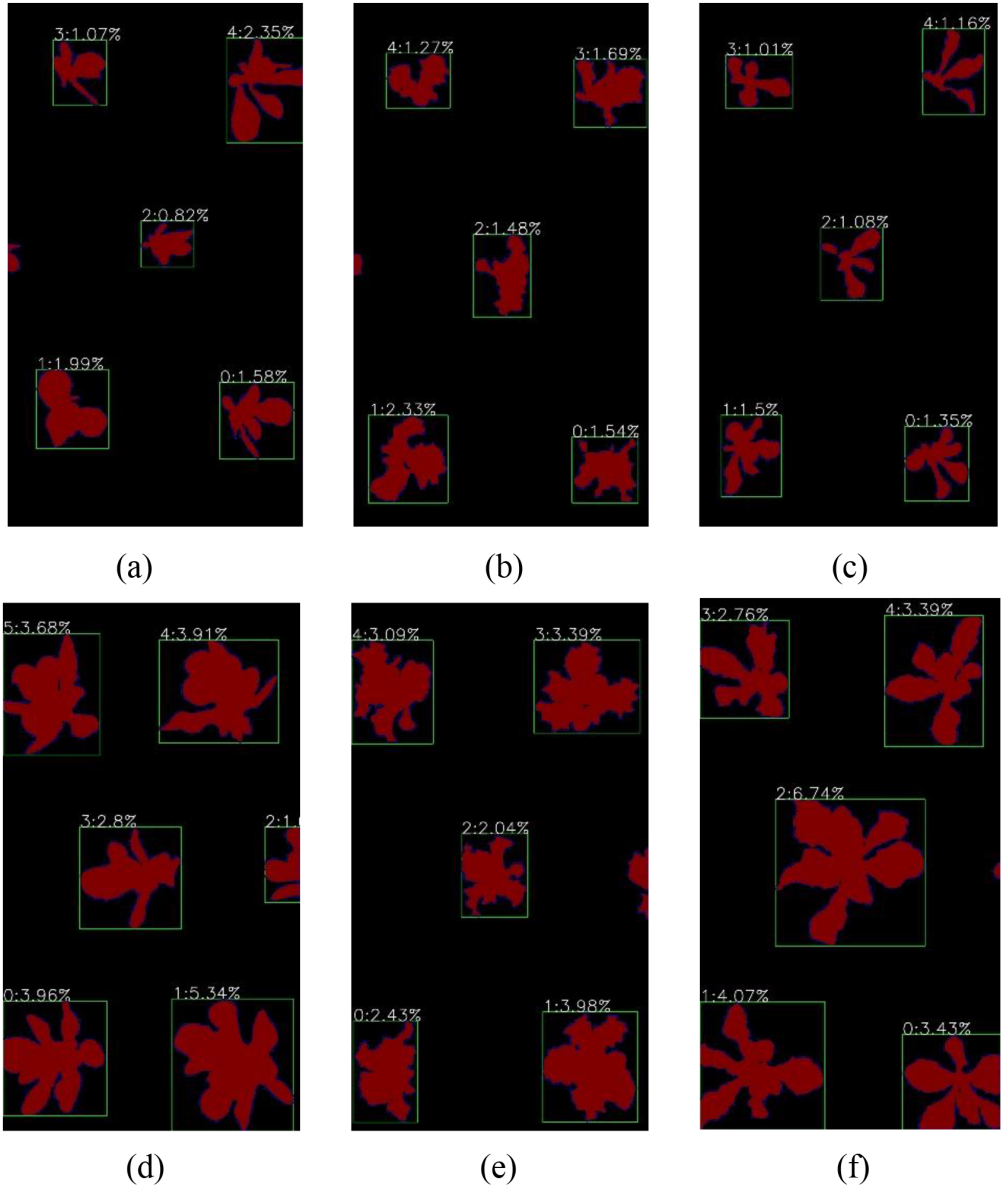
Analysis of individual plant growth differences. **(a)** S1-V1 **(b)** S1-V2 **(c)** S1-V3 **(d)** S2-V1 **(e)** S2-V2 **(f)** S2-V3.

Accurate acquisition of canopy coverage or leaf area is one of the important indicators for evaluating plant growth. Currently, some studies obtain the canopy ground cover of crop groups for field scenes and compare the advantages and disadvantages among various sensors. However, the process of obtaining canopy coverage with RGB digital camera is based on the pixel discrimination standard of (green – red)/(green + Red) > 0 (Deery et al., 2021). This method can greatly improve the speed of detection in practical applications, but there may be a certain gap with the deep learning method in accuracy (Yang et al., 2020; Sadashivan et al., 2021). Some studies may be limited by the cultivation mode (pot or pipe cultivation) and mainly estimate the leaf area of a single plant, but in the actual large-scale planting process, the canopy coverage or leaf area of a single plant has certain limitations, which is difficult to reflect the growth status of the same batch of the group plants (Rangarajan and Purushothaman, 2020; Mohammadi et al., 2021; Trivedi and Gupta, 2021; Bhagat et al., 2022). Therefore, this study fully considered the individual growth differences in the group plants and conducted a statistical analysis of the individual growth differences of the group plants. Among them, for the group plants in the same monitoring area, we used the ratio of the number of pixels of the target plant to the number of pixels of the whole viewing area to evaluate the growth difference of individual plants. The specific results are shown in **Figure 9**.

As shown in **Figure 9**, we selected partial images of S1 and S2 in the early growth stages of three varieties of lettuce for analysis of individual plant growth differences. It can be found that individual plant growth differences of different varieties of lettuce occurred in the early growth stages. As shown in figures (a), (b), and (c), the individual canopy coverage of multi-variety lettuce plants in the S1 stage can be basically divided into three levels: 0%-1%, 1%-2%, and 2%-3%. Similarly, as shown in figures (d), (e), and (f), it is found that the individual canopy coverage of multi-variety lettuce plants in the S2 stage can be generally divided into three levels: 2%-3%, 3%-4%, and 4%-7%. The reason may be that the root development degree of different plants is inconsistent in the process of seedling, resulting in further differences in nutrient absorption, and finally reflected in individual growth differences. Therefore, this study evaluated the growth of large-scale crops by obtaining the canopy coverage of the group lettuce of greenhouse cultivation.

### 4.2 The advantages and challenges of practical application

In Section 4.1, this study confirmed the fact that individual plants have growth differences. The canopy coverage of the group plants was obtained by using the semantic segmentation method, and the growth status of lettuce of different varieties and different growth stages was monitored (see Section 3.5 for details). Although we have improved PSPNet by combining ECA attention mechanism without increasing the size of the model, challenges of identifying the dynamic process of different varieties of lettuce of the whole life cycle in practical application are still existed. Therefore, in order to improve the segmentation of PSPNet on the group lettuce plants in the whole growth stage, this study proposed a new attention mechanism called CAS. CAS reinforces the features of the PSPNet backbone, which significantly improves MIoU indicators, ensuring accurate segmentation of PSPNet in practice. Considering the lightweight deployment requirements of the model in actual planting scenarios, this study replaced the original backbone ResNet50 of PSPNet with MobileNetv3 and InceptionNeXt for lightweight improvement. The results showed that PSPNet based on MobileNetv3 achieved a good balance between model performance and model complexity. MobileNetV3 and InceptionNeXt both have fewer parameters and smaller model-size, which helps reduce storage and computing resource consumption and improve model efficiency. However, compared to InceptionNeXt, MobileNetV3 has fewer parameters and a more lightweight model.

**Figure 10:**
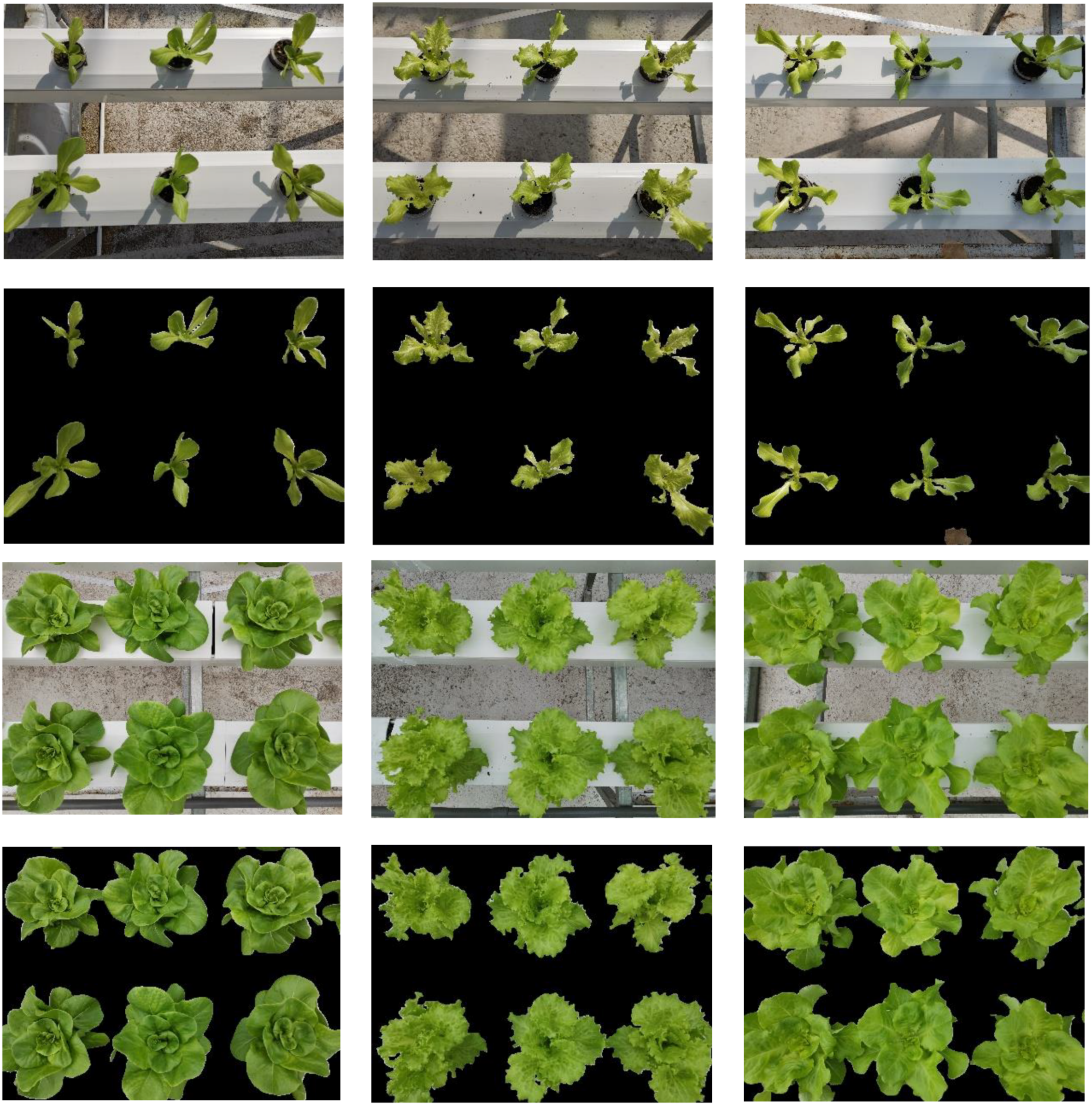
Segmentation verification of the group lettuce in pipeline cultivation.

It is well known that the segmentation method based on deep learning shows significant advantages in model accuracy and generalization performance compared with the traditional segmentation method. Besides benefiting from the advantages of the deep learning model, it also relies on a large number of manually labeled sample data. To explore the generalization and applicability of the proposed segmentation model in other planting scenarios, this study collected images of the group lettuce from partial pipeline cultivation for validation, as shown in **Figure 10**. It was found that the proposed semantic segmentation model displays good generalization performance in other planting scenarios. However, considering the annotation of segment datasets is extremely time-consuming, semi-supervised or unsupervised learning seems promising in subsequent research.

In the practical application of segmentation models, different agricultural scenarios and crop types face different challenges. The field environment is usually complex, including different soil types, lighting conditions, weather conditions, crop growth stages, etc. These variations may lead to different appearances and textures of crops, thereby decreasing segmentation precision. The internal environment of facility greenhouses is relatively stable but still faces the issue of uneven lighting. High density planting and staggered cultivation of multiple crops in greenhouses may raise higher demands on the accuracy of semantic segmentation algorithms. Crop types vary in characteristics, such as shape, size, and color. Even the same crop type may exhibit different appearances due to different growth stages and environments, making it very difficult to design a universal semantic segmentation model. The proposed method in this study is limitedly applicable to the scene of near-point growth state monitoring such as facility greenhouse and plant factory rather than large-scale scenes such as a field. Thus it is necessary to expand corresponding image datasets and fine-tune the model to meet the application needs in field. Finally, there is no leaf occlusion between plants in the early stage of crop growth, but inevitably occurs in the late stage of crop growth. In this case, the accurate acquisition of plant canopy coverage may also need to consider the spacing between plants, which is very easy to achieve in scenarios such as facility greenhouses and plant factories.

## 5 Conclusion

Canopy coverage is one of the core indicators for measuring lettuce growth, and the automated acquisition of this indicator is crucial for dynamically monitoring the growth status of lettuce. However, individual growth differences among the group plants at different growth stages cause difficulties in accurately evaluating the growth status of the group lettuce based on the canopy coverage of individuals. Focusing on greenhouse hydroponic lettuce, this study improves the model from two perspectives: improving model precision and reducing model-size. Firstly, an attention mechanism CAS is proposed to improve PSPNet, which considers channel features, spatial features, and axial features simultaneously, addressing issues such as high similarity between some plant stems and background, as well as differences in edge growth of different lettuce varieties. The MIoU can reach 0.9832. Meanwhile, in order to meet the deployment requirements of the model in practical planting scenarios, this study improved PSPNet by replacing a lightweight backbone and establishing a lightweight semantic segmentation model based on MobileNetv3-PSPNet, with an MIoU of 0.9717. Besides, a group lettuce canopy coverage acquisition system was constructed based on PyQt5 to facilitate the automatic analysis of dynamic growth changes for the further exploration. When Loaded into the system, this pre-trained model enables automatic calculation of canopy coverage of the group lettuce. This study provides a feasible, automated, and low-cost method and platform for real-time monitoring of crop growth in practical planting scenarios such as facility greenhouses and plant factories, which can be applied to other crops for growth management in the future. At present, this proposed method is only suitable for small-scale, near-ground hydroponic lettuce growth monitoring. Further data acquisition and model fine-tuning will be required to popularize the proposed model to various crops and scenes in the future. Therefore, it is suggested to further expand the scope of application of the model across different varieties and planting scenarios, and obtain more evaluation parameters of crop growth using this method to enrich the model function and improve the model performance.

## Data Availability

The raw data supporting the conclusions of this article will be made available by the authors, without undue reservation.

## References

[B1] Adams J. Qiu Y. Xu Y. Schnable J. C. (2020). Plant segmentation by supervised machine learning methods. Plant Phenome J. 3, 1–11. doi: 10.1002/ppj2.20001

[B2] Badrinarayanan V. Kendall A. Cipolla R. (2017). SegNet: A deep convolutional encoder-decoder architecture for image segmentation. IEEE Trans. Pattern Anal. Mach. Intell. 39, 2481–2495. doi: 10.1109/TPAMI.2016.2644615, PMID: 28060704

[B3] Bhagat S. Kokare M. Haswani V. Hambarde P. Kamble R. (2022). Eff-UNet++: A novel architecture for plant leaf segmentation and counting. Ecol. Inform. 68, 101583. doi: 10.1016/j.ecoinf.2022.101583

[B4] Cao S. Xu B. Zhou W. Zhou L. Zhang J. Zheng Y. . (2025). The blessing of Depth Anything: An almost unsupervised approach to crop segmentation with depth-informed pseudo labeling. Plant Phenomics 7, 100005. doi: 10.1016/j.plaphe.2025.100005, PMID: 41415954 PMC12709960

[B5] Chang C. L. Chung S. C. Fu W. L. Huang C. C. (2021). Artificial intelligence approaches to predict growth, harvest day, and quality of lettuce (Lactuca sativa L.) in a IoT-enabled greenhouse system. Biosyst. Eng. 212, 77–105. doi: 10.1016/j.biosystemseng.2021.09.015

[B6] Chaurasia A. Culurciello E. (2018). LinkNet: Exploiting encoder representations for efficient semantic segmentation. 2017 IEEE Vis. Commun. Image Process. VCIP 2017 2018, 1–4. doi: 10.1109/VCIP.2017.8305148

[B7] Chen L. C. Zhu Y. Papandreou G. Schroff F. Adam H. (2018). Encoder-decoder with atrous separable convolution for semantic image segmentation. Lect. Notes Comput. Sci. (including Subser. Lect. Notes Artif. Intell. Lect. Notes Bioinformatics) 11211 LNCS, 833–851. doi: 10.1007/978-3-030-01234-2_49

[B8] Das S. Kar S. Sekh A. A. (2021). “ FGrade: A large volume dataset for grading tomato freshness quality,” in Computer vision and image processing. CVIP 2020. Communications in computer and information science. Eds. Singh S. K. Roy P. Raman B. Nagabhushan P. ( Springer Singapore, Singapore), 455–466.

[B9] Das S. Mondal P. Quraishi M. I. Kar S. Sekh A. A. (2022). “ Freshness quality detection of Tomatoes Using Computer Vision,” in Artificial Intelligence. ISAI 2022. Communications in Computer and Information. Eds. Turki T. Ghosh T. K. Joardar S. Barman S. ( Springer Nature Switzerland, Cham), 243–255. doi: 10.1007/978-3-031-22485-0_22

[B10] Deery D. M. Smith D. J. Davy R. Jimenez-Berni J. A. Rebetzke G. J. James R. A. (2021). Impact of varying light and dew on ground cover estimates from active NDVI, RGB, and LiDAR. Plant Phenomics 2021, 9842178. doi: 10.34133/2021/9842178, PMID: 34250506 PMC8240513

[B11] Du R. Ma Z. Xie P. He Y. Cen H. (2023). PST: Plant segmentation transformer for 3D point clouds of rapeseed plants at the podding stage. ISPRS J. Photogramm. Remote Sens. 195, 380–392. doi: 10.1016/j.isprsjprs.2022.11.022

[B12] Fan Y. Chen Y. Chen X. Zhang H. Liu C. Duan Q. (2021). Estimating the aquatic-plant area on a pond surface using a hue-saturation-component combination and an improved Otsu method. Comput. Electron. Agric. 188, 106372. doi: 10.1016/j.compag.2021.106372

[B13] G. J. B. Gopi E. S. (2021). An hierarchical approach for automatic segmentation of leaf images with similar background using kernel smoothing based Gaussian process regression. Ecol. Inform. 63, 101323. doi: 10.1016/j.ecoinf.2021.101323

[B14] Gangwar A. Rani G. Singh Dhaka V. (2025). Tomato TransDeepLab: A Robust Framework for Tomato Leaf Segmentation, Disease Severity prediction, and crop loss estimation. IEEE Access 13, 170147–170160. doi: 10.1109/ACCESS.2025.3611307

[B15] Gou F. van Ittersum M. K. Couëdel A. Zhang Y. Wang Y. van der Putten P. E. L. . (2018). Intercropping with wheat lowers nutrient uptake and biomass accumulation of maize, but increases photosynthetic rate of the ear leaf. AoB Plants 10, 1–15. doi: 10.1093/aobpla/ply010, PMID: 29479410 PMC5817965

[B16] Guerra Ibarra J. P. Cuevas de la Rosa F. J. Hernandez Vidales J. R. (2025). Evaluation of the effectiveness of the UNet model with different backbones in the semantic segmentation of tomato leaves and fruits. Horticulturae 11, 1–19. doi: 10.3390/horticulturae11050514

[B17] Guo M. H. Lu C. Z. Hou Q. Liu Z. N. Cheng M. M. Hu S. M. (2022). SegNeXt: rethinking convolutional attention design for semantic segmentation. Adv. Neural Inf. Process. Syst. 35, 1–15. doi: 10.48550/arXiv.2209.08575

[B18] Guo R. Qu L. Niu D. Li Z. Yue J. (2021). LeafMask: towards greater accuracy on leaf segmentation. Proc. IEEE Int. Conf. Comput. Vis. 2021, 1249–1258. doi: 10.1109/ICCVW54120.2021.00145

[B19] Ho J. Kalchbrenner N. Weissenborn D. Salimans T. (2019). Axial attention in multidimensional transformers. Comput. Vis. Pattern Recognit. 1–11. doi: 10.48550/arXiv.1912.12180

[B20] Howard A. G. Zhu M. Chen B. Kalenichenko D. Wang W. Weyand T. . (2017). MobileNets: efficient convolutional neural networks for mobile vision applications. Proc. IEEE Comput. Soc Conf. Comput. Vis. Pattern Recognit. doi: 10.48550/arXiv.1704.04861

[B21] Jiang Y. Li C. Paterson A. H. Sun S. Xu R. Robertson J. (2018). Quantitative analysis of cotton canopy size in field conditions using a consumer-grade RGB-D camera. Front. Plant Sci. 8. doi: 10.3389/fpls.2017.02233, PMID: 29441074 PMC5797632

[B22] Kamilaris A. Prenafeta-Boldú F. X. (2018). Deep learning in agriculture: A survey. Comput. Electron. Agric. 147, 70–90. doi: 10.1016/j.compag.2018.02.016

[B23] Kang C. Mu X. Novaski Seffrin A. Di Gioia F. He L. (2025). A recursive segmentation model for bok choy growth monitoring with Internet of Things (IoT) technology in controlled environment agriculture. Comput. Electron. Agric. 230, 109866. doi: 10.1016/j.compag.2024.109866

[B24] Keller K. (2018). Soybean leaf coverage estimation with machine learning and thresholding algorithms for field phenotyping. Available online at: https://www.plant-phenotyping.org/lw_resource/datapool/systemfiles/elements/files/6b71e293-949c-11e8-8a88-dead53a91d31/current/document/0032.pdf.

[B25] Kong X. Liu T. Chen X. Jin X. Li A. Yu J. (2024). Efficient crop segmentation net and novel weed detection method. Eur. J. Agron. 161, 127367. doi: 10.1016/j.eja.2024.127367

[B26] Kubota C. Meng C. Masoud S. Son Y. Tronstad R. (2019). Advanced technologies for large- scale plant factories — Integration of industrial and systems engineering crop production. Plant Factory Using Artificial Light. 353–362. doi: 10.1016/B978-0-12-813973-8.00033-6

[B27] Lee U. Chang S. Putra G. A. Kim H. Kim D. H. (2018). An automated, high-throughput plant phenotyping system using machine learning-based plant segmentation and image analysis. PloS One 13, 1–17. doi: 10.1371/journal.pone.0196615, PMID: 29702690 PMC5922545

[B28] Li J. Pu F. Chen H. Xu X. Yu Y. (2024). Crop segmentation of unmanned aerial vehicle imagery using edge enhancement network. IEEE Geosci. Remote Sens. Lett. 21, 1–5. doi: 10.1109/LGRS.2024.3358983

[B29] Li D. Shi G. Li J. Chen Y. Zhang S. Xiang S. . (2022). PlantNet: A dual-function point cloud segmentation network for multiple plant species. ISPRS J. Photogramm. Remote Sens. 184, 243–263. doi: 10.1016/j.isprsjprs.2022.01.007

[B30] Li Z. Xiang L. Sun J. Liao D. Xu L. Wang M. (2025). A multi-level knowledge distillation for enhanced crop segmentation in precision agriculture. Agric. 15, 1–25. doi: 10.3390/agriculture15131418

[B31] Li S. Yan Z. Guo Y. Su X. Cao Y. Jiang B. . (2021). SPM-IS: An auto-algorithm to acquire a mature soybean phenotype based on instance segmentation. Crop J 10(5), 1412–1423. doi: 10.1016/j.cj.2021.05.014

[B32] Lin G. Milan A. Shen C. Reid I. (2017). RefineNet: Multi-path refinement networks for high-resolution semantic segmentation. Proc. - 30th IEEE Conf. Comput. Vis. Pattern Recognition CVPR 2017 2017, 5168–5177. doi: 10.1109/CVPR.2017.549

[B33] Liu Z. Lin Y. Cao Y. Hu H. Wei Y. Zhang Z. . (2021). Swin transformer: Hierarchical vision transformer using shifted windows. IEEE Int. Conf. Comput. Vis., 9992–10002. doi: 10.1109/ICCV48922.2021.00986

[B34] Lu Y. Young S. Wang H. Wijewardane N. (2022). Robust plant segmentation of color images based on image contrast optimization. Comput. Electron. Agric. 193, 106711. doi: 10.1016/j.compag.2022.106711

[B35] Ma J. Du K. Zheng F. Zhang L. Gong Z. Sun Z. (2018). A recognition method for cucumber diseases using leaf symptom images based on deep convolutional neural network. Comput. Electron. Agric. 154, 18–24. doi: 10.1016/j.compag.2018.08.048

[B36] Makanza R. Zaman-Allah M. Cairns J. E. Magorokosho C. Tarekegne A. Olsen M. . (2018). High-throughput phenotyping of canopy cover and senescence in maize field trials using aerial digital canopy imaging. Remote Sens. 10(2), 300. doi: 10.3390/rs10020330, PMID: 33489316 PMC7745117

[B37] Marani R. Milella A. Petitti A. Reina G. (2021). Deep neural networks for grape bunch segmentation in natural images from a consumer-grade camera. Precis. Agric. 22, 387–413. doi: 10.1007/s11119-020-09736-0

[B38] Masuda T. (2021). Leaf area estimation by semantic segmentation of point cloud of tomato plants. Proc. IEEE Int. Conf. Comput. Vis. 2021, 1381–1389. doi: 10.1109/ICCVW54120.2021.00159

[B39] Mohammadi V. Minaei S. Mahdavian A. R. Khoshtaghaza M. H. Gouton P. (2021). Estimation of Leaf Area in Bell Pepper Plant using Image Processing techniques and Artificial Neural Networks. 2021 IEEE Int. Conf. Signal Image Process. Appl., 173–178. doi: 10.1109/icsipa52582.2021.9576778

[B40] Mu Y. Wang H. Hu J. Sun Y. Zhu C. Zhu H. . (2026). PCSNet: A lightweight semantic segmentation model for low-altitude remote sensing mixed crop segmentation for rapid acquisition of planting information. Comput. Electron. Agric. 241, 111297. doi: 10.1016/j.compag.2025.111297

[B41] Nekrasov V. Shen C. Reid I. (2019). Light-weight refinenet for real-time semantic segmentation. Br. Mach. Vis. Conf. 2018 BMVC 2018, 1–19. doi: 10.48550/arXiv.1810.03272

[B42] Nikbakhsh N. Baleghi Y. Agahi H. (2021). A novel approach for unsupervised image segmentation fusion of plant leaves based on G-mutual information. Mach. Vis. Appl. 32. doi: 10.1007/s00138-020-01130-0

[B43] Olaf R. Philipp F. Thomas B. (2015). UNet: convolutional networks for biomedical image segmentation. ArXiv, abs/1505.0. doi: 10.1109/ACCESS.2021.3053408

[B44] Pan H. Hong Y. Sun W. Jia Y. (2023). Deep dual-resolution networks for real-time and accurate semantic segmentation of traffic scenes. IEEE Trans. Intell. Transp. Syst. 24, 3448–3460. doi: 10.1109/TITS.2022.3228042

[B45] Paszke A. Chaurasia A. Kim S. Culurciello E. (2016). ENet: A deep neural network architecture for real-time semantic segmentation. Proc. IEEE Comput. Soc Conf. Comput. Vis. Pattern Recognit., 1–10. doi: 10.48550/arXiv.1606.02147

[B46] Peng C. Zhang X. Yu G. Luo G. Sun J. (2017). Large kernel matters - Improve semantic segmentation by global convolutional network. Proc. - 30th IEEE Conf. Comput. Vis. Pattern Recognition CVPR 2017 2017, 1743–1751. doi: 10.1109/CVPR.2017.189

[B47] Picon A. Eguskiza I. Galan P. Gomez-Zamanillo L. Romero J. Klukas C. . (2025). Crop-conditional semantic segmentation for efficient agricultural disease assessment. Artif. Intell. Agric. 15, 79–87. doi: 10.1016/j.aiia.2025.01.002

[B48] Quan T. M. Hildebrand D. G. C. Jeong W. K. (2021b). FusionNet: A deep fully residual convolutional neural network for image segmentation in connectomics. Front. Comput. Sci. 3. doi: 10.3389/fcomp.2021.613981

[B49] Quan P. Lou Y. Lin H. Liang Z. Di S. (2021a). Research on fast identification and location of contour features of electric vehicle charging port in complex scenes. IEEE Access PP, 1. doi: 10.1109/ACCESS.2021.3092210

[B50] Rahim U. F. Utsumi T. Mineno H. (2021). Comparison of grape flower counting using patch-based instance segmentation and density-based estimation with convolutional neural networks. Int. Symp. Artif. Intell. Robot. 2021, 72. doi: 10.1117/12.2605670

[B51] Rangarajan A. K. Purushothaman R. (2020). A vision based crop monitoring system using segmentation techniques. Adv. Electr. Comput. Eng. 20, 89–100. doi: 10.4316/AECE.2020.02011

[B52] Sadashivan S. Bhattacherjee S. S. Priyanka G. Pachamuthu R. Kholova J. (2021). Fully automated region of interest segmentation pipeline for UAV based RGB images. Biosyst. Eng. 211, 192–204. doi: 10.1016/j.biosystemseng.2021.08.032

[B53] Shelhamer E. Long J. Darrell T. (2017). Fully convolutional networks for semantic segmentation. IEEE Trans. Pattern Anal. Mach. Intell. 39, 640–651. doi: 10.1109/TPAMI.2016.2572683, PMID: 27244717

[B54] Shu M. Li Q. Ghafoor A. Zhu J. Li B. Ma Y. (2023). Using the plant height and canopy coverage to estimation maize aboveground biomass with UAV digital images. Eur. J. Agron. 151, 126957. doi: 10.1016/j.eja.2023.126957

[B55] Singh A. K. Ganapathysubramanian B. Sarkar S. Singh A. (2018). Deep learning for plant stress phenotyping: trends and future perspectives. Trends Plant Sci. 23, 883–898. doi: 10.1016/j.tplants.2018.07.004, PMID: 30104148

[B56] Tian Z. Ma W. Yang Q. Duan F. (2021). Application status and challenges of machine vision in plant factory — A review Application status and challenges of machine vision in plant factory — A review. Inf. Process. Agric. 9(2), 195–211. doi: 10.1016/j.inpa.2021.06.003

[B57] Trivedi M. Gupta A. (2021). Automatic monitoring of the growth of plants using deep learning-based leaf segmentation. Int. J. Appl. Sci. Eng. 18, 1–9. doi: 10.6703/IJASE.202106_18(2).003

[B58] Wang Q. Wu B. Zhu P. Li P. Zuo W. Hu Q. (2020). ECA-Net: Efficient channel attention for deep convolutional neural networks. Proc. IEEE Comput. Soc Conf. Comput. Vis. Pattern Recognit., 11531–11539. doi: 10.1109/CVPR42600.2020.01155

[B59] Woo S. Park J. Lee J. Y. Kweon I. S. (2018). CBAM: Convolutional block attention module. Eur. Conf. Comput. Vis. 11211, 3–19. doi: 10.1007/978-3-030-01234-2_1

[B60] Xu X. Li H. Yin F. Xi L. Qiao H. Ma Z. . (2020). Wheat ear counting using K − means clustering segmentation and convolutional neural network. Plant Methods 16, 1–13. doi: 10.1186/s13007-020-00648-8, PMID: 32782453 PMC7412807

[B61] Yang K. Zhong W. Li F. (2020). Leaf segmentation and classification with a complicated background using deep learning. Agronomy 10(11), 1721. doi: 10.3390/agronomy10111721

[B62] Yoosefzadeh-Najafabadi M. Tulpan D. Eskandari M. (2021). Application of machine learning and genetic optimization algorithms for modeling and optimizing soybean yield using its component traits. PloS One 16, 1–18. doi: 10.1371/journal.pone.0250665, PMID: 33930039 PMC8087002

[B63] Yu C. Wang J. Peng C. Gao C. Yu G. Sang N. (2018). BiSeNet: Bilateral segmentation network for real-time semantic segmentation. Lect. Notes Comput. Sci. (including Subser. Lect. Notes Artif. Intell. Lect. Notes Bioinformatics) 11217 LNCS, 334–349. doi: 10.1007/978-3-030-01261-8_20

[B64] Yu W. Zhou P. Yan S. Wang X. (2023). InceptionNeXt: when inception meets convNeXt. arXiv 2303, 16900. doi: 10.48550/arXiv.2303.16900

[B65] Zabawa L. Kicherer A. Klingbeil L. Töpfer R. Kuhlmann H. Roscher R. (2020). Counting of grapevine berries in images via semantic segmentation using convolutional neural networks. ISPRS J. Photogramm. Remote Sens. 164, 73–83. doi: 10.1016/j.isprsjprs.2020.04.002

[B66] Zenkl R. Timofte R. Kirchgessner N. Roth L. Hund A. Van Gool L. . (2022). Outdoor plant segmentation with deep learning for high-throughput field phenotyping on a diverse wheat dataset. Front. Plant Sci. 12. doi: 10.3389/fpls.2021.774068, PMID: 35058948 PMC8765702

[B67] Zhang X.-D. (2020). “ Machine learning,” in Matrix Algebra Approach to Artificial intelligence ( Springer Singapore, Singapore), 223–440. doi: 10.1007/978-981-15-2770-8_6

[B68] Zhang C. Si Y. Lamkey J. Boydston R. A. Garland-Campbell K. A. Sankaran S. (2018a). High-throughput phenotyping of seed/seedling evaluation using digital image analysis. Agronomy 8, 1–14. doi: 10.3390/agronomy8050063

[B69] Zhang Z. Zhang X. Peng C. Xue X. Sun J. (2018b). ExFuse: Enhancing feature fusion for semantic segmentation. Lect. Notes Comput. Sci. 11214, 273–288. doi: 10.1007/978-3-030-01249-6_17

[B70] Zhao Y. Li T. Wen W. Lu X. Yang S. Fan J. . (2025). YOMASK: An instance segmentation method for high-throughput phenotypic platform lettuce images. Comput. Electron. Agric. 230, 109868. doi: 10.1016/j.compag.2024.109868

[B71] Zhao H. Shi J. Qi X. Wang X. Jia J. (2017). Pyramid scene parsing network. Proc. - 30th IEEE Conf. Comput. Vis. Pattern Recognition CVPR 2017 2017, 6230–6239. doi: 10.1109/CVPR.2017.660

[B72] Zheng Z. Yuan J. Yao W. Yao H. Liu Q. Guo L. (2024). Crop classification from drone imagery based on lightweight semantic segmentation methods. Remote Sens. 16(21), 4099. doi: 10.3390/rs16214099

[B73] Zhu C. Miao T. Xu T. Yang T. Li N. (2020). Stem-leaf segmentation and phenotypic trait extraction of maize shoots from three-dimensional point cloud. Proc. IEEE Int. Conf. Comput. Vis. 1–26. doi: 10.48550/arXiv.2009.03108

